# The need for tailoring school-based physical activity interventions: preliminary insights into body weight and cross-country differences from the DELICIOUS project

**DOI:** 10.3389/fpubh.2025.1675893

**Published:** 2025-12-18

**Authors:** Achraf Ammar, Mohamed Aly, Khaled Trabelsi, Tania Abril-Mera, Liwa Masmoudi, Noha El-Gyar, Amira M. Shalaby, Haitham Jahrami, Waqar Husain, Piotr Zmijewski, Evelyn Frias-Toral, Giuseppe Grosso, Wolfgang I. Schöllhorn, Osama Abdelkarim

**Affiliations:** 1 Department of Training and Movement Science, Institute of Sport Science, Johannes Gutenberg University of Mainz, Mainz, Germany; 2 Research Laboratory, Molecular Bases of Human Pathology, LR19ES13, Faculty of Medicine of Sfax, University of Sfax, Sfax, Tunisia; 3 Interdisciplinary Laboratory in Neurosciences, Physiology, and Psychology: Physical Activity, Health, and Learning (LINP2), UFR STAPS, Paris Nanterre University, Nanterre, France; 4 Faculty of Sport Sciences, Assiut University, Assiut, Egypt; 5 ESLSCA University Egypt, Giza, Egypt; 6 Research Laboratory: Education, Motricity, Sport and Health, EM2S, LR19JS01, High Institute of Sport and Physical Education of Sfax, University of Sfax, Sfax, Tunisia; 7 High Institute of Sport and Physical Education of Sfax, University of Sfax, Sfax, Tunisia; 8 Department of Movement Sciences and Sports Training, School of Sport Science, The University of Jordan, Amman, Jordan; 9 School of Medicine, Universidad Católica de Santiago de Guayaquil, Guayaquil, Ecuador; 10 Faculty of Medicine, Department of Pediatric, Assiut University, Assiut, Egypt; 11 Government Hospitals, Manama, Bahrain; 12 Department of Psychiatry, College of Medicine and Health Sciences, Arabian Gulf University, Manama, Bahrain; 13 Department of Humanities, COMSATS University Islamabad, Islamabad, Pakistan; 14 Department of Biochemistry, Gdansk University of Physical Education and Sport, Gdansk, Poland; 15 Escuela de Medicina, Universidad Espíritu Santo, Samborondón, Ecuador; 16 Division of Research, Texas State University, San Marcos, TX, United States; 17 Department of Biomedical and Biotechnological Sciences, University of Catania, Catania, Italy

**Keywords:** physical fitness, exercise intervention, Mediterranean region, cross-cultural study, youth health promotion, early adolescents, children, anthropometrics

## Abstract

**Background:**

Although school-based physical activity (PA) programs are recognized for enhancing children’s health-related fitness (HRF), limited evidence exists on how responsiveness varies by country and body weight status. Within the framework of the DELICIOUS project, this study analyzed cross-country variations in anthropometric and health-related fitness (HRF) changes among children with normal weight, overweight, and obesity who participated in a standardized school-based PA intervention.

**Methods:**

Over 900 children aged 8–14 years from Egypt, Lebanon, Italy, Portugal, and Spain participated in a standardized six-month PA-program. Anthropometric measures (weight, height, and BMI) and physical fitness components (sprint, jump, strength, endurance, and coordination) were assessed before and after the intervention. Intervention effects were analyzed using repeated measures and factorial ANOVA models to examine interactions between time, country, and body weight category.

**Results:**

The intervention showed the greatest anthropometric effectiveness in Egypt and Spain, where children with overweight and obesity experienced weight stabilization and BMI reductions, significant among groups with obesity (−4% in Egypt; −2% in Spain). In contrast, Lebanon and Italy exhibited slight but significant increases in BMI among participants with normal and overweight. Regarding physical performance, the intervention led to significant improvements across countries, particularly in coordination and cardiovascular endurance. The most comprehensive gains were observed among children with overweight, with Egypt showing improvements across all fitness outcomes, and Lebanon and Portugal improving in all except sprint. Among normal-weight, participants in Lebanon, Egypt, and Portugal improved in 4 to 5 out of 6 fitness tests, whereas those in Spain and Italy improved in only 2 to 3. Children with obesity exhibited the lowest responsiveness overall, with Egypt, Italy, Spain, and Portugal showing improvements in only 1 to 2 outcomes.

**Conclusion:**

The standardized PA intervention yielded promising, yet heterogeneous HRF changes among Mediterranean children, differing by country and weight status. These findings highlight the importance of adapting school-based PA programs to local sociocultural contexts and individual profiles. In particular, vulnerable groups such as children with obesity may require tailored, multicomponent interventions that extend beyond standardized PA to include nutritional education, psychological support, and culturally adapted strategies to optimize outcomes and promote sustained engagement.

## 1 Introduction

Physical activity (PA) is a cornerstone of healthy development during childhood and adolescence, offering well-established physical, mental, and cognitive benefits (1). Regular PA improves cardiorespiratory fitness, muscular strength, coordination, metabolic health, and bone development, while also supporting cognitive functions such as attention, memory, and academic performance (1–5). To achieve these benefits, international guidelines recommend that children and adolescents aged 5–17 engage in at least 60 min of moderate-to-vigorous PA daily, emphasizing aerobic activities and including vigorous, muscle- and bone-strengthening exercises at least three times per week (6, 7). Conversely, physical inactivity increases the risk of overweight, obesity, and non-communicable diseases even from early childhood (8–10). Alarmingly, based on 298 surveys from 146 countries, more than 80% of adolescents fail to meet current PA guidelines, with prevalence of insufficient activity ranging from 79% in high-income to 85% in low-income countries (11). This situation was further exacerbated by the COVID-19 pandemic, which increased sedentary behavior and restricted access to structured PA opportunities (12–15).

Despite their traditional association with active lifestyles and healthy dietary patterns (16, 17), Mediterranean countries now exhibit some of the highest rates of childhood overweight and obesity in Europe (18, 19). This paradox is largely attributed to rapid cultural and socioeconomic transitions, including increased screen time, urbanization, reduced active transport, and growing adherence to westernized, energy-dense diets (11, 20–22). These shifts have contributed to a marked decline in health-related fitness (HRF) indicators among youth in Mediterranean regions (22).

School-based PA interventions have proven effective in improving HRF, particularly when combining aerobic, strength, and skill-based components in structured, age-appropriate formats (23–26). Schools provide equitable access to all children, but intervention outcomes may vary according to protocol design, baseline fitness, age, sex, and especially weight status (24, 27–29). Compared with their normal-weight peers, children with overweight may demonstrate greater relative improvements in body composition and motor skills (30). Conversely, despite promising evidence supporting the benefits of PA, particularly aerobic-based programs, in improving cardiovascular and metabolic health among children with overweight or obesity (31–33), this more vulnerable population may exhibit reduced responsiveness, especially in high-impact or weight-bearing activities due to biomechanical limitations, lower aerobic capacity, and diminished self-efficacy (34, 35). In such cases, resistance training may represent a more feasible and motivating alternative, as children with obesity often possess greater absolute muscle mass and strength, facilitating participation. Moreover, cultural, environmental, and curricular variations between countries may influence participation and effectiveness, underscoring the need for cross-national comparative studies (24, 36).

The DELICIOUS project, a multicountry initiative supported by the EU PRIMA program, sought to counteract the growing burden of physical inactivity by promoting healthy diet and PA habits among school-aged children in Egypt, Lebanon, Italy, Spain, and Portugal. A standardized, school-based PA intervention was implemented and assessed in 937 participants aged 8–14 years. Our previously published analysis demonstrated significant improvements in several HRF components, particularly coordination, endurance, and muscular strength, with substantial variation in effectiveness by country and age group (24). Early adolescents generally exhibited greater gains than younger participants, while among countries, Lebanon and Portugal showed the most pronounced improvements, and more modest effects were observed in Spain and Italy. The intervention was also effective in preventing excessive weight gain and improving BMI in specific subgroups, notably Egyptian adolescents (24).

While these findings offered valuable insights into the cross-country effectiveness of PA interventions, they did not account for the potential moderating effect of body-weight status. This aspect is particularly relevant given the rising prevalence of childhood overweight and obesity across Mediterranean countries (10, 18, 19, 22) and emerging evidence that children with higher BMI may respond differently to structured PA (27, 28, 30, 34, 35, 37). Previous studies examining HRF responses across weight groups have typically used binary classifications (normal weight vs. overweight) or limited HRF domains (30, 37). For example, Katanic et al. (37) assessed coordination and locomotor skills, while Piri et al. (30) focused on agility and sprint performance, often neglecting muscular strength and endurance. This limited scope restricts a comprehensive understanding of how PA interventions affect the full range of HRF across weight categories. Moreover, cross-national comparative studies remain scarce (24), particularly those systematically evaluating whether and how children with overweight or obesity respond to a standardized intervention across diverse Mediterranean contexts.

Taken together, these gaps highlight the need for a more comprehensive investigation that disaggregates intervention outcomes by multiple weight categories and examines a broad spectrum of HRF indicators within a multicountry framework. The present study therefore provides the first large-scale cross-national analysis of how children with different weight statuses (normal weight, overweight, and obesity) respond to a standardized school-based PA intervention across five Mediterranean countries. Specifically, we examined the interaction between body-weight category and country of residence in shaping changes in key anthropometric (weight, height, BMI) and physical performance outcomes (sprint, strength, power, endurance, coordination), aiming to identify which subgroups benefit most and to inform development of culturally adapted, weight-sensitive school-based strategies.

## 2 Methods

### 2.1 Participants

Participants were recruited from five countries participating in the DELICIOUS consortium: Egypt, Italy, Lebanon, Portugal, and Spain. The DELICIOUS project, which has been described in detail elsewhere (38, 39), is a multinational initiative designed to promote adherence to the Mediterranean diet and enhance physical activity among school-aged children and adolescents. A pragmatic recruitment strategy was utilized, prioritizing the inclusion of as many eligible participants as possible within the project’s operational and logistical framework. Public schools were selected on the basis of their willingness to participate and practical accessibility, and most eligible students in those schools were invited to join. This approach allowed for the use of convenience sampling, balancing methodological considerations with the realities of conducting fieldwork in diverse international settings. The final sample comprised 937 children and early adolescents aged between 8 and 14 years, who were recruited from public schools located in urban centers of five Mediterranean cities: Assiut (Egypt), Lisbon (Portugal), Beirut (Lebanon), Cordoba (Spain), and Giugliano in Campania (Italy). Participants were excluded if they were absent from 14 or more intervention sessions (≈20% of the total sessions implemented), if they failed to attend either the pre- or post-intervention testing session, or if they did not complete the full set of anthropometric and health-related fitness assessments. These criteria ensured sufficient exposure to the intervention and complete data for analysis. Prior to participation, written informed consent was obtained from the parents or legal guardians of all the children. The study received ethical approval from the ethics committee of Mondragon University (IEB-20230704) and was conducted in accordance with the principles of the Declaration of Helsinki.

### 2.2 Physical activity intervention program

A standardized, school-based physical activity program was developed within the framework of the DELICIOUS project and implemented across all participating Mediterranean countries. The program was informed by evidence-based models of “Build Our Kids’ Success (BOKS)” (40) and was designed to improve key components of physical fitness including speed, agility, strength, endurance, and coordination. The activities were age-appropriate, inclusive, and noncompetitive, with a particular focus on engaging children who are typically less active. The intervention spanned 6 months and consisted of structured sessions aimed at developing functional movement skills. Each session followed a consistent format that included a warm-up, targeted skill instruction, physical exercises, and game-based activities to reinforce the skills practiced. Sessions were conducted three times per week, each lasting between 40 and 45 min. Delivering the same program across culturally diverse settings allowed for the assessment of cross-country differences in outcomes. All sites followed a standardized session frequency, duration, and structure, supported by shared instructor briefing materials to ensure consistent delivery. While local attendance records were maintained, harmonized fidelity data (e.g., session delivery logs and instructor training records) were not systematically captured across all sites, precluding quantitative cross-country comparisons. Participant adherence was evaluated using the predefined exclusion threshold of absence from ≥14 sessions (≈20%), alongside completion of both pre- and post-intervention testing sessions.

### 2.3 International physical performance test profile

The International Physical Performance Test Profile (IPPTP) is a standardized and validated test battery designed to assess HRF in children and adolescents. It offers a comprehensive evaluation of key physical fitness components, including speed, agility, strength, endurance, and coordination, making it especially suitable for cross-cultural research (23, 41–44). The IPPTP includes eight test items that reflect the five core dimensions of physical fitness: endurance, strength, speed, coordination, and flexibility. These tests are widely recognized as reliable and valid field-based measures of physical fitness. For this study, six specific test items were selected from the IPPTP to capture the primary aspects of children’s HRF, which were chosen for their high validity and cross-cultural applicability. Speed was assessed using the 20-meter dash, which recorded the time taken to sprint over a 20-meter distance. Agility and coordination were evaluated through the Sideways Jumping test (SJ), in which participants performed as many lateral jumps as possible within 15 s. Upper-body muscular strength and endurance were measured using the Push-Up test (PU), based on the number of push-ups completed in 40 s, while core strength was assessed via the Sit-Up test (SU), which involves the number of sit-ups performed within the same time frame. Lower-body strength was evaluated using the Standing Long Jump (SLJ), where the distance jumped from a standing position was recorded in centimeters. Finally, cardiovascular endurance was assessed with the 6-min run, by measuring the total distance covered during the six-minute period. All the assessments were conducted under standardized conditions to ensure procedural consistency and enhance the reliability of the results across the different cultural and national contexts. Further technical details regarding the administration and scoring of the test items are available in the official IPPTP manuals (43).

### 2.4 Anthropometry

Anthropometric measurements were collected prior to the physical fitness assessments. Standing height was measured to the nearest 0.1 cm using a stadiometer (Seca 213, Seca GmbH & Co. KG, Hamburg, Germany), and body weight was assessed using a digital body composition scale (Tanita BF-350 Total Body Composition Analyzer, Amsterdam, the Netherlands). Body mass index (BMI) was subsequently calculated as weight in kilograms divided by height in meters squared (kg/m^2^).

#### 2.4.1 Classification of body weight categories

In this study, we classified participants’ weight status using WHO BMI-for-age growth standards, which account for age- and sex-specific differences in pediatric populations (45). BMI-for-age z-scores were calculated on the basis of WHO reference charts, with classifications as follows: normal weight (z-score between −2 and +1), overweight (z-score > + 1 to ≤ + 2), and obesity (z-score > + 2). This method provides a standardized and internationally recognized approach for assessing childhood weight status, ensuring comparability with global research. All groupings used in the analyses were based exclusively on WHO BMI-for-age z-scores, whose corresponding BMI values vary by age and sex across the currently studied 8–14-year range.

### 2.5 Statistical analysis

All statistical analyses were conducted using SPSS (version 25). The normality of the data was verified using the Shapiro–Wilk test. Analyses were structured to examine time (pre–post) × country × weight-status interactions on raw data, followed by country × weight-status comparisons of delta (Δ%) changes, allowing evaluation of within-subject improvement patterns and between-group differences. Accordingly, a three-way mixed ANOVA (2 levels for time: pre- and post-intervention × 3 levels for body-weight category: normal weight, overweight, and obesity × 5 levels for country) was conducted to evaluate the effects of the intervention program on the primary outcomes (BMI and health-related fitness variables) and the secondary outcomes (body weight and height) across different body-weight categories and countries using pre- and post-intervention data. Additionally, a two-way ANOVA (3 levels for body weight categories × 5 levels for countries) was applied to assess the effects of the intervention program on the same parameters using percentage changes from pre- to post-intervention. Post-hoc differences were investigated using the Bonferroni test to identify pairwise comparisons between groups. The effect size was calculated using partial eta squared (ηp^2^) to evaluate the magnitude of change. Statistical significance was set *a priori* at *p* < 0.05 for all analyses.

## 3 Results

Data from 920 participants who completed both pre- and post-intervention assessments and met adherence criteria were analyzed. The baseline prevalence of normal weight, overweight, and obesity differed significantly between countries (*p* < 0.001). Overweight status was the most prevalent category overall (≈ 44–54%), whereas obesity was relatively higher in Egypt and Spain (≈ 29–33%) and lowest in Portugal (≈ 9%). Despite small numerical shifts (e.g., a few transitions between overweight and normal or obese categories), no statistically significant redistribution of BMI-for-age z-score–based categories was observed across the full sample (*p* > 0.05).

### 3.1 Effects of the intervention program on body weight, height, and BMI across different body weight categories and countries

Three-way repeated-measures ANOVA (Table 1) revealed significant time × weight-category × country interactions for weight and BMI, whereas height showed only significant time × country and weight-category × country interactions. Main effects of time and weight category were significant for all anthropometric variables, and country effects emerged for weight and height. *Post-hoc* comparisons indicated clear cross-country contrasts. BMI decreased significantly only in Egypt and Spain among children with obesity, while Lebanon and Italy showed small but significant BMI increases among normal-weight and overweight children (*p* < 0.05). Weight increased significantly across most countries; however, in Egypt, this increase was observed only among children with normal weight (*p* < 0.05). Height rose across all groups, consistent with normal growth patterns, though a time × country interaction indicated smaller height gains in Italy, where the increase reached significance only among children with overweight and obesity (*p* < 0.05).

**Table 1:** Body weight, height, and body mass index (BMI) at pre- and post-intervention categorized by body weight categories and countries.

Variables	BMI z-score category	Country of living	Means±SD		Main effect			Significant interaction
			Pre	Post	Time effect	Weight category effect	Country effect	
Weight (kg)	Normal weight	Egypt	32.4 ± 5.3	33.6 ± 5.4*	F_(1, 905)_ = 301.59; *p* < 0.001; η_p_^2^ = 0.25	F_(2, 905)_ = 427.57; *p* < 0.001; η_p_^2^ = 0.49	F_(4, 905)_ = 26.3; *p* < 0.001; η_p_^2^ = 0.1	Weight Category × Country (*F*_(8, 905)_ = 2.01; *p* = 0.043; η_p_^2^ = 0.02) Time × Weight Category (*F*_(2, 905)_ = 12.65; *p* < 0.001; η_p_^2^ = 0.03) Time × Country (*F*_(4, 905)_ = 22.51; *p* < 0.001; η_p_^2^ = 0.09) Time × Weight Category × Country (*F*_(8, 905)_ = 3.29; *p* = 0.001; η_p_^2^ = 0.03)
		Lebanon	30.8 ± 6.5	32.8 ± 7.5*				
		Italy	29.9 ± 3.5	31.6 ± 4.2*				
		Spain	36.4 ± 5.7	38.2 ± 6.3*				
		Portugal	31.5 ± 5.8	32.8 ± 6.7*				
	Overweight	Egypt	44 ± 7.4 N	44.8 ± 7.4 N				
		Lebanon	43.7 ± 7.3 N	46.1 ± 8.2*N				
		Italy	36.9 ± 5.2 NEL	38.1 ± 5.5*EL				
		Spain	50 ± 8.4 NELI	51 ± 8.6*NELI				
		Portugal	43.4 ± 8.7NIS	44.9 ± 9.4*NIS				
	Obesity	Egypt	57.4 ± 9.2 NO	56.6 ± 8.9NO				
		Lebanon	58.9 ± 12.1 NO	61.2 ± 12.2*NO				
		Italy	51.1 ± 8.5 NOL	51.4 ± 8.4 NOL				
		Spain	61.2 ± 12.1 NOI	61.8 ± 12.3 NOI				
		Portugal	55.3 ± 9.7NO	56.2 ± 9.1 NO				
Height (cm)	Normal weight	Egypt	144.9 ± 9.8	147.1 ± 9.7*	F_(1, 905)_ = 1446.1; *p* < 0.001; η_p_^2^ = 0.62	F_(2, 905)_ = 36.45; *p* < 0.001; η_p_^2^ = 0.07	F_(4, 905)_ = 38.93; *p* < 0.001; η_p_^2^ = 0.15	Weight Category × Country (F_(8, 905)_ = 2.65; *p* = 0.007; η_p_^2^ = 0.02) Time × Country (F_(4, 905)_ = 32.57; *p* < 0.001; η_p_^2^ = 0.13)
		Lebanon	141.5 ± 12.7	143.5 ± 12.9*				
		Italy	139.5 ± 7.1	140 ± 7				
		Spain	152.7 ± 11.2LI	155 ± 11.1*LI				
		Portugal	142.1 ± 11.1S	144.3 ± 11.2*S				
	Overweight	Egypt	152.4 ± 11.1 N	154.3 ± 11.1*N				
		Lebanon	150.9 ± 11 N	153 ± 10.8*N				
		Italy	140 ± 7.6EL	140.8 ± 7.4*EL				
		Spain	160.4 ± 11.5ELI	162.6 ± 11.4*ELI				
		Portugal	151.1 ± 12.9NIS	153.1 ± 12.7*NIS				
	Obesity	Egypt	152.5 ± 9.9	154.6 ± 9.9*				
		Lebanon	155.2 ± 12.1 N	157 ± 11.8*N				
		Italy	145 ± 7.9 L	145.9 ± 7.5*L				
		Spain	157.7 ± 10.9I	159.9 ± 10.9*I				
		Portugal	151.1 ± 9.1	152.6 ± 8.8*				
BMI (kg.m-2)	Normal weight	Egypt	15.3 ± 1.1	15.5 ± 1.1	F_(1, 905)_ = 11.21; *p* < 0.001; η_p_^2^ = 0.01	F_(2, 905)_ = 1170.92; *p* < 0.001; η_p_^2^ = 0.72	F_(4, 905)_ = 1.11; *p* = 0.35; η_p_^2^ = 0	Time × Weight Category (F_(2, 905)_ = 27.71; *p* < 0.001; η_p_^2^ = 0.06) Time × Country (F_(4, 905)_ = 32; *p* < 0.001; η_p_^2^ = 0.12) Time × Weight Category × Country (F_(8, 905)_ = 4.52; *p* < 0.001; η_p_^2^ = 0.04)
		Lebanon	15.2 ± 1.1	15.7 ± 1.4*				
		Italy	15.3 ± 1	16.1 ± 1.4*				
		Spain	15.5 ± 1	15.8 ± 1.3				
		Portugal	15.5 ± 1	15.6 ± 1.3				
	Overweight	Egypt	18.8 ± 1.3 N	18.7 ± 1.3 N				
		Lebanon	19.1 ± 1.2 N	19.5 ± 1.6*N				
		Italy	18.7 ± 1.4 N	19.2 ± 1.5*N				
		Spain	19.3 ± 1.4 N	19.2 ± 1.5 N				
		Portugal	18.8 ± 1.4 N	19 ± 1.5 N				
	Obesity	Egypt	24.6 ± 2.6NO	23.6 ± 2.3*NO				
		Lebanon	24.2 ± 3NO	24.7 ± 3.2 NO				
		Italy	24.2 ± 3NO	24.1 ± 3.1 NO				
		Spain	24.5 ± 3NO	24 ± 2.9*NO				
		Portugal	24.1 ± 2.4 NO	24 ± 2.3 NO				

BMI, body mass index; *significant difference compared to “Pre” at *p* < 0.05; N significant difference compared to “Normal weight” at *p* < 0.05; O significant difference compared to “Overweight” at *p* < 0.05; E significant difference compared to “Egypt” at *p* < 0.05; L significant difference compared to “Lebanon” at *p* < 0.05; I significant difference compared to “Italy” at *p* < 0.05; S significant difference compared to “Spain” at *p* < 0.05.

As expected, children with obesity showed higher weight and BMI than those with overweight, who in turn exceeded normal-weight peers (*p* < 0.05). Between-country contrasts showed that Spanish participants displayed the highest mean anthropometric values, particularly in the overweight and obesity groups. Owing to the standardized BMI-for-age z-score used for weight classification, BMI did not significantly differ between countries within any weight category.

**Figure 1:**
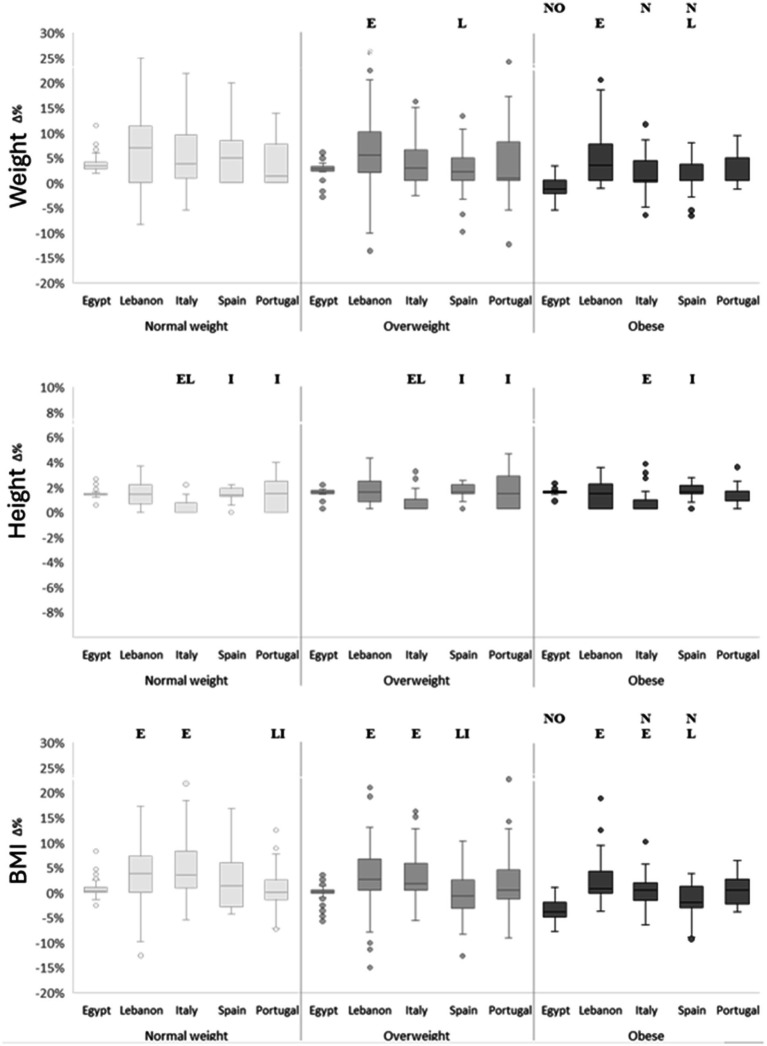
Percentage change (Δ%) in weight, height and body mass index (BMI) according to weight categories and countries. N significant difference compared to “Normal weight” at *p* < 0.05; O significant difference compared to “Overweight” at *p* < 0.05; E significant difference compared to “Egypt”; I significant difference compared to “Italy” at *p* < 0.05; L significant difference compared to “Lebanon” at *p* < 0.05.

Analysis of percentage changes (Figure 1) confirmed a significant weight-category × country interaction for BMI (*F*(8, 905) = 2.83, *p* = 0.004, ηp^2^ = 0.02). Only Egypt and Spain presented negative changes in the overweight (−0.8%) and obese groups (−4% and −2%, respectively). This reduction in BMI resulted in significant differences compared with most other countries, particularly Lebanon and Italy, across all weight categories (*p* < 0.05). Lebanese children with overweight and obesity showed the greatest relative weight gain (≈ 5.5 and 4%, respectively), whereas Italian children presented the smallest height change (< 1%). Overall, children with obesity exhibited the lowest relative change in both weight and BMI, particularly in Egypt, Italy, and Spain, indicating partial weight stabilization compared with their normal-weight counterparts (*p* < 0.05).

### 3.2 Effects of the intervention program on physical fitness performance across different age groups and countries

Three-way repeated-measures ANOVA (time × weight-category × country) revealed a significant triple interaction only for SU, while time × country effects were significant for most variables except sprint (Table 2). Time × weight-category effects appeared for sprint and SLJ.

**Table 2:** Physical fitness performance at pre- and post-intervention categorized by body weight categories and countries.

Variables	BMI z-score category	Country of living	Means ± SD		Main effect			Significant interaction
			Pre	Post	Time effect	Weight category effect	Country effect	
Sprint (sec)	Normal weight	Egypt	4.16 ± 0.5	3.91 ± 0.33	F_(1, 905)_ = 56.45; *p* < 0.001; η_p_^2^ = 0.06	F_(2, 905)_ = 7; *p* < 0.001; η_p_^2^ = 0.02	F_(4, 905)_ = 12.14; *p* < 0.001; η_p_^2^ = 0.05	Time × Weight Category (F_(2, 905)_ = 3.11; *p* = 0.045; η_p_^2^ = 0.01)
		Lebanon	4.26 ± 0.56	4.16 ± 0.51				
		Italy	4.1 ± 0.48	4.04 ± 0.33				
		Spain	4.5 ± 0.45	4.39 ± 0.45E				
		Portugal	4.2 ± 0.48	4.12 ± 0.51				
	Overweight	Egypt	4.32 ± 0.52	3.91 ± 0.4*				
		Lebanon	4.26 ± 0.52	4.06 ± 0.53				
		Italy	4.28 ± 0.58	4.1 ± 0.45				
		Spain	4.36 ± 0.55	4.23 ± 0.54E				
		Portugal	4.35 ± 0.61	3.97 ± 0.57*				
	Obesity	Egypt	4.4 ± 0.59	4.19 ± 0.51				
		Lebanon	4.47 ± 0.57	4.23 ± 0.46				
		Italy	4.31 ± 0.58	4.15 ± 0.4				
		Spain	4.59 ± 0.56	4.42 ± 0.55				
		Portugal	4.27 ± 0.61	4.09 ± 0.43				
SLJ (cm)	Normal weight	Egypt	137.9 ± 27.1	147.9 ± 19.6	F_(1, 905)_ = 153.26; *p* < 0.001; η_p_^2^ = 0.14	F_(2, 905)_ = 6.9; *p* = 0.001; η_p_^2^ = 0.02	F(4, 905) = 2.04; *p* = 0.087; ηp^2^ = 0.01	Time × Weight Category (F_(2, 905)_ = 5.13; *p* = 0.006; η_p_^2^ = 0.01) Time × Country (F_(4, 905)_ = 5; *p* < 0.001; η_p_^2^ = 0.02)
		Lebanon	136.5 ± 32.3	153 ± 24.7*				
		Italy	139.2 ± 36	150.4 ± 20.6				
		Spain	136.3 ± 24.9	147.3 ± 24.7				
		Portugal	135.9 ± 27.4	155 ± 21.2*				
	Overweight	Egypt	141.6 ± 25.8	153.8 ± 20.8*				
		Lebanon	127.7 ± 27.6	153.4 ± 28.3*				
		Italy	137.3 ± 30	146.8 ± 20.6				
		Spain	143.9 ± 28.8 L	152.3 ± 26.3				
		Portugal	132.5 ± 29.9	166.7 ± 28*I				
	Obesity	Egypt	132.5 ± 23.5	144.2 ± 21.5				
		Lebanon	126.1 ± 28.6	138.5 ± 23.3				
		Italy	138.7 ± 24.5	143.7 ± 18.6				
		Spain	131 ± 27.5	139.1 ± 25.8				
		Portugal	138.9 ± 31.6	150.8 ± 30.4				
JSW (n)	Normal weight	Egypt	27.5 ± 6.6	31.4 ± 5*	F_(1, 905)_ = 574.6; *p* < 0.001; η_p_^2^ = 0.39	F_(2, 905)_ = 0.53; *p* = 0.589; η_p_^2^ = 0	F_(4, 905)_ = 15.56; *p* < 0.001; η_p_^2^ = 0.06	Time × Country (F_(4, 905)_ = 15.69; *p* < 0.001; η_p_^2^ = 0.06)
		Lebanon	28.8 ± 8.6	32.3 ± 6.7*				
		Italy	31.3 ± 7.5	36.7 ± 6.6*				
		Spain	22.4 ± 7.3LI	30.7 ± 7.4*				
		Portugal	28 ± 7.7	37.5 ± 6.2*ELS				
	Overweight	Egypt	27.7 ± 7.5	32 ± 5.4*				
		Lebanon	27.2 ± 6.5	31.2 ± 5.8*				
		Italy	28.6 ± 7.3	34.8 ± 5.8*				
		Spain	25.7 ± 7.3	31.7 ± 7.8*				
		Portugal	26.6 ± 7	37.9 ± 5.6*ELS				
	Obesity	Egypt	27.7 ± 6.4	30.9 ± 4.5				
		Lebanon	25.6 ± 6.5	30.6 ± 4.9*				
		Italy	28.8 ± 7.6	34 ± 5*				
		Spain	25.9 ± 7.9	31.5 ± 7.3*				
		Portugal	28.8 ± 7.1	36.8 ± 6.6*				
PU (n)	Normal weight	Egypt	14.8 ± 3.9	18.3 ± 2.8*	*F*_(1, 905)_ = 344.8; *p* < 0.001; η_p_^2^ = 0.28	F_(2, 905)_ = 1.49; *p* = 0.226; η_p_^2^ = 0	F_(4, 905)_ = 4.49; *p* = 0.001; η_p_^2^ = 0.02	Time × Country (F_(4, 905)_ = 4; *p* = 0.003; η_p_^2^ = 0.02)
		Lebanon	14.3 ± 4.8	19 ± 4.8*				
		Italy	15.9 ± 5.3	19.4 ± 5.3				
		Spain	16.4 ± 4.8	19.8 ± 3.4				
		Portugal	15.1 ± 4.8	19.6 ± 4.7*				
	Overweight	Egypt	14.1 ± 3.9	18.2 ± 3.3*				
		Lebanon	13.8 ± 4.7	18.7 ± 4.9*				
		Italy	14.5 ± 4.9	19.9 ± 5.1*				
		Spain	16.8 ± 6.4EL	20.4 ± 5.2*				
		Portugal	14.7 ± 4.5	19.5 ± 4.8*				
	Obesity	Egypt	15.1 ± 4	17.6 ± 4.5				
		Lebanon	12.8 ± 4.4	19.6 ± 5.7*				
		Italy	15.9 ± 5.8	18.1 ± 4.2				
		Spain	15.1 ± 6.5	18.5 ± 5.1*				
		Portugal	15 ± 6.1	18.3 ± 3.8				
SU (n)	Normal weight	Egypt	21.8 ± 6.4	26.6 ± 4.9*	F_(1, 905)_ = 200.7; *p* < 0.001; η_p_^2^ = 0.18	F_(2, 905)_ = 2.35; *p* = 0.096; η_p_^2^ = 0.01	F_(4, 905)_ = 15.15; *p* < 0.001; η_p_^2^ = 0.06	Time × Country (F_(4, 905)_ = 3.41; *p* = 0.009; η_p_^2^ = 0.01) Time × Weight Category × Country (F_(8, 905)_ = 2.27; *p* = 0.021; η_p_^2^ = 0.02)
		Lebanon	20.5 ± 7.5	25.2 ± 6.5*				
		Italy	21.9 ± 9.7	27.8 ± 7.6*				
		Spain	24.8 ± 10	27.1 ± 9.7				
		Portugal	22 ± 6.7	25.1 ± 7.8				
	Overweight	Egypt	21.1 ± 6.6	25.4 ± 6.4*				
		Lebanon	19.8 ± 6.5	24.8 ± 6.6*				
		Italy	22.7 ± 7.1	27.5 ± 4.7*				
		Spain	29.4 ± 12.6ELI	31.2 ± 12.3EL				
		Portugal	20.2 ± 6.6S	28.7 ± 8.3*				
	Obesity	Egypt	21.3 ± 7.1	24.7 ± 7.3				
		Lebanon	18.2 ± 7.1	23.5 ± 4.9*				
		Italy	22.4 ± 6.4	26.7 ± 5.6				
		Spain	25.6 ± 11 L	28 ± 10.2				
		Portugal	22.1 ± 7.4	25.9 ± 5.8				
Run (m)	Normal weight	Egypt	915 ± 157	1,164 ± 153*	F_(1, 905)_ = 1540.04; *p* < 0.001; η_p_^2^ = 0.63	F_(2, 905)_ = 2.43; *p* = 0.089; η_p_^2^ = 0.01	F_(4, 905)_ = 5.29; *p* < 0.001; η_p_^2^ = 0.02	Weight Category × Country (F_(8, 905)_ = 1.96; *p* = 0.048; η_p_^2^ = 0.02) Time × Country (F_(4, 905)_ = 2.53; *p* = 0.039; η_p_^2^ = 0.01)
		Lebanon	929 ± 188	1,186 ± 144*				
		Italy	1,013 ± 219	1,238 ± 173*				
		Spain	879 ± 177	1,134 ± 119*				
		Portugal	904 ± 193	1,255 ± 159*				
	Overweight	Egypt	889 ± 162	1,150 ± 152*				
		Lebanon	884 ± 162	1,182 ± 151*				
		Italy	946 ± 179	1,186 ± 144*				
		Spain	945 ± 197	1,175 ± 167*				
		Portugal	888 ± 194	1,216 ± 180*				
	Obesity	Egypt	902 ± 184	1,169 ± 166*				
		Lebanon	866 ± 181	1,179 ± 107*				
		Italy	946 ± 182	1,187 ± 150*				
		Spain	835 ± 188O	1,108 ± 145*				
		Portugal	952 ± 183	1,153 ± 182*				

BMI, body mass index; PU, Push-Ups; SU, Sit-Ups; SLJ, Standing Long Jump; JSW, Jump Sideways; *significant difference compared to “Pre” at p < 0.05; N significant difference compared to “Normal weight” at *p* < 0.05; O significant difference compared to “Overweight” at *p* < 0.05; E significant difference compared to “Egypt” at *p* < 0.05; L significant difference compared to “Lebanon” at *p* < 0.05; I significant difference compared to “Italy” at *p* < 0.05; S significant difference compared to “Spain” at *p* < 0.05.

Overall, physical-fitness gains were evident across most parameters, with overweight groups showing the most consistent improvements across countries. Sprint performance improved significantly over time only in children with overweight from Egypt and Portugal (*p* < 0.05). SLJ improved significantly in Lebanese and Portuguese children with normal weight and overweight, and in Egyptian children with overweight (*p* < 0.05). JSW increased significantly from pre- to post-intervention across most countries and weight categories, except in Egyptian children with obesity (*p* < 0.05). PU improved significantly among children with overweight in all countries (*p* < 0.05), with additional gains in Lebanese children with normal weight and obesity, Egyptian and Portuguese children with normal weight, and Spanish children with obesity (all p < 0.05). SU improved significantly among children with normal weight and overweight in Egypt, Lebanon, and Italy, with further gains in Portuguese children with overweight and Lebanese children with obesity (*p* < 0.05). Endurance (6-min run) improved significantly in all weight categories across all countries (*p* < 0.05). Additionally, post-hoc contrasts indicated generally comparable results across weight categories and countries, except for a few specific differences: Egyptian children outperformed their Spanish peers in post-intervention sprint performance (normal-weight and overweight groups); Portuguese children with overweight surpassed their Italian counterparts in post-intervention SLJ; Portuguese children with normal weight and overweight outperformed their Egyptian, Lebanese, and Spanish peers in post-intervention JSW; and Spanish children with normal weight achieved the highest SU scores both pre- and post-intervention (all *p* < 0.05). PU and endurance results followed similar overall patterns.

**Figure 2:**
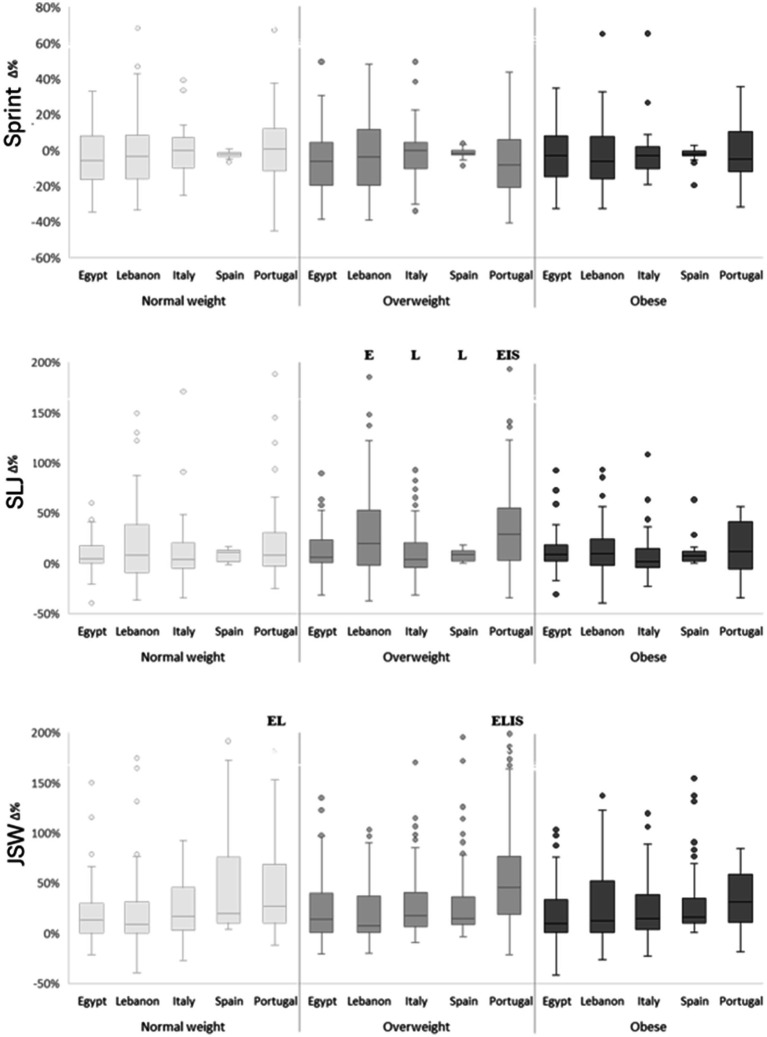
Percentage change (Δ%) in sprint, standing long jump (SLJ) and jump sideways (JSW) performance according to weight categories and countries. E significant difference compared to “Egypt” at *p* < 0.05; L significant difference compared to “Lebanon” at *p* < 0.05; I significant difference compared to “Italy” at *p* < 0.05; S significant difference compared to “Spain” at *p* < 0.05.

**Figure 3:**
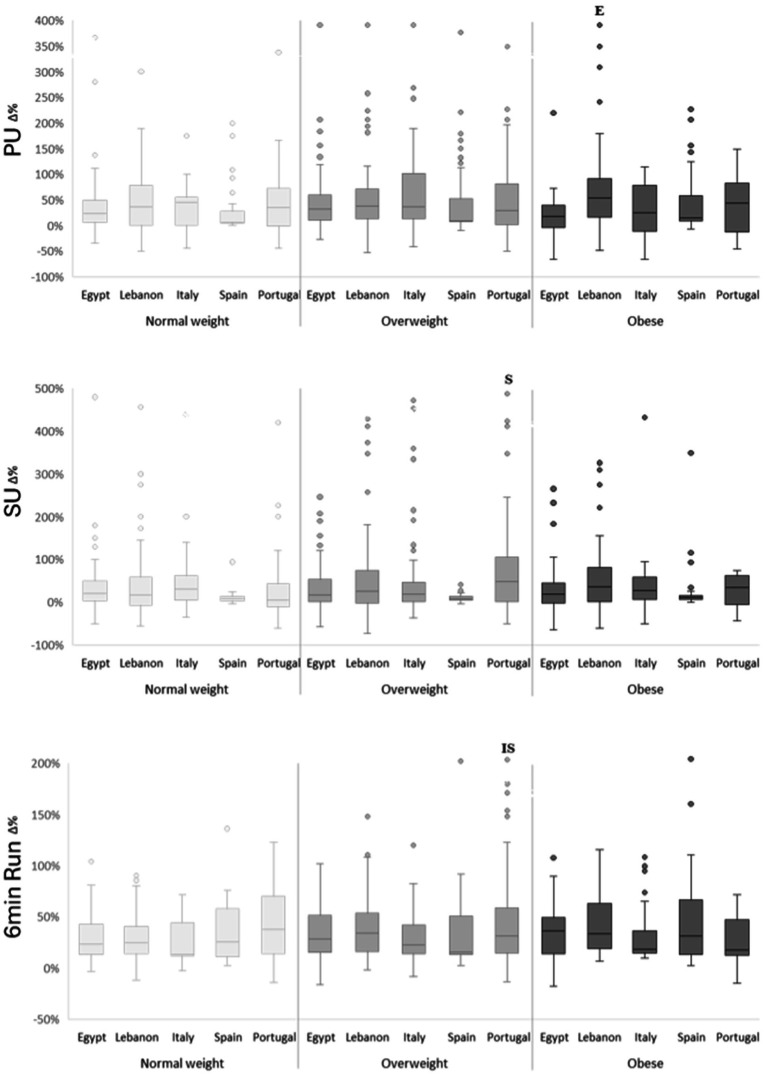
Percentage change (Δ%) in push-ups (PU), sit-ups (SU) and 6 min running performances according to weight categories and countries. E significant difference compared to “Egypt” at *p* < 0.05; I significant difference compared to “Italy” at *p* < 0.05; S significant difference compared to “Spain” at *p* < 0.05.

Figures 2, 3 summarize percentage changes. Two-way ANOVA on percentage change revealed main effects of weight category for sprint (*F*(2, 905) = 3.09, *p* = 0.046, ηp^2^ = 0.007) and SLJ (F(2, 905) = 4.24, *p* = 0.01, ηp^2^ = 0.009), and main effects of country for most tests (*F*(4, 905) ≈ 2.39–8.99; *p* = 0.05–< 0.001; ηp^2^ ≈ 0.01–0.04), except sprint. There were no significant weight-category × country interactions for any performance variable. Post-hoc contrasts showed that, for most parameters, the majority of significant between-country differences were observed in the overweight group. Lebanese (~20%) and Portuguese (~26%) children with overweight recorded the largest SLJ improvements, outperforming peers from Egypt, Spain, and Italy (*p* < 0.05). JSW percentage gains were highest in Portugal for both normal-weight (~34%) and overweight (~43%) children; these exceeded those observed in Egypt and Italy in the normal-weight group and in all countries in the overweight group (*p* < 0.05). For SU, Portuguese children with overweight (~33%) improved more than their Spanish peers (~21%; *p* < 0.05). Endurance followed a similar trend, with Portuguese children with overweight (~42%) outperforming Spanish (~6%) and Italian (~20%) peers (*p* < 0.05). Among children with obesity, the only significant between-country difference was observed in PU, where Lebanese participants (+53%) improved more than their Egyptian counterparts (+16%; *p* < 0.05). No significant within-country differences were detected among weight categories for relative performance gains (*p* > 0.05), indicating that the intervention produced comparable effects across weight groups.

## 4 Discussion

This study aimed to evaluate the outcomes of a standardized school-based physical activity (PA) intervention across five Mediterranean countries (Egypt, Lebanon, Italy, Spain, and Portugal) considering variations by body weight categories. The overall prevalence of normal weight, overweight, and obesity remained stable, with no significant redistribution between categories despite minor individual transitions. This finding aligns with expectations for short-term, school-based PA interventions, which typically emphasize BMI stabilization and gradual improvements in adiposity rather than rapid categorical shifts (32, 46). Our main results further revealed significant interactions between weight status and country, suggesting differential patterns of change in anthropometric parameters and physical performance outcomes across these groups. Across countries, the most favorable trends were observed in Egypt, where children with overweight or obesity maintained stable weight and reduced BMI, while Spain showed modest BMI reductions in similar groups. Improvements in physical performance were most pronounced among children with overweight, particularly in Egypt, whereas those with obesity showed limited gains across most fitness outcomes. These comparative findings should, however, be interpreted descriptively, as pre–post changes cannot be uniquely attributed to the program in the absence of a control group.

### 4.1 Anthropometric outcomes: body weight, height, and BMI

Our findings regarding anthropometric measures indicated notable variations in intervention responsiveness by body weight category and country. An expected growth-related increase in height and weight occurred throughout the intervention period across most countries and weight categories, consistent with natural child growth trajectories (47). However, notably, Egyptian children with overweight and obesity showed no significant weight gain, a finding with important implications for obesity management interventions. Stabilization of weight in populations with overweight is considered a favorable outcome, particularly in this developmental age, and can reflect improved body composition through increased muscle mass and decreased fat percentage (27, 28, 32, 35, 46, 48, 49). Similar stabilization findings in children with obesity across other countries, except for Lebanon, further support potential effectiveness in managing unhealthy weight gain through structured PA programs. The BMI results offer further nuanced insights. Lebanon and Italy displayed significant increases in BMI among normal and overweight categories, suggesting possible issues related to program implementation, adherence, or concurrent dietary patterns in these regions, warranting further investigation (24, 50–52). Conversely, Egypt and Spain exhibited BMI reductions among children with overweight (≈1%) and obesity (−4% and −2%, respectively), reaching statistical significance in populations with obesity, and highlighting the intervention’s potential to reduce obesity risk effectively. Notably, the results in the Egyptian and Spanish cohorts align with previous research reporting greater BMI reductions among Iranian children with overweight, compared to their normal-weight peers, following a 12-week physical activity program (30). Various biological mechanisms can explain the observed effectiveness. Regular PA increases energy expenditure, promoting an energy deficit necessary for reducing body fat (32, 46). Additionally, previous studies, including pediatric interventions, have reported enhanced metabolic efficiency following physical activity or combined lifestyle programs, with improvements in lipid and glucose metabolism and insulin sensitivity, thereby reducing adiposity, especially visceral fat, which is strongly linked to obesity-related complications (48, 49, 53–56). Exercise-induced adaptations in skeletal muscle, also observed in pediatric populations, enhance resting metabolic rate and mitochondrial function (57, 58), further supporting long-term energy balance and healthy weight management. Moreover, regular PA stimulates beneficial hormonal responses, such as increased secretion of growth hormone and catecholamines, which promote lipolysis and reduce fat storage (59–61). Similar BMI stabilization or reductions have been previously associated with successful PA interventions (32, 46), reinforcing the importance of structured PA programs tailored to specific country contexts and population characteristics.

At both pre- and post-intervention measurement, Spain consistently recorded the highest anthropometric values (i.e., weight and height), while Italy displayed the lowest across the majority of weight categories, a disparity consistent with previous regional reports highlighting nutritional, lifestyle, and socio-cultural differences across Mediterranean countries (10, 24, 62, 63). Lifestyle factors, including dietary patterns, levels of PA outside the structured school environment, and sedentary behaviors, significantly influence anthropometric outcomes. For example, differences in adherence to traditional Mediterranean dietary practices, which emphasize the consumption of fruits, vegetables, whole grains, and healthy fats, have been shown to vary considerably among Mediterranean countries, potentially explaining regional discrepancies in anthropometric measurements (16, 17, 50, 52). Moreover, variations in extracurricular PA opportunities and built environments, such as the availability of recreational spaces or active commuting options, could further amplify differences in anthropometric values (51). These between-country differences may partly reflect broader dietary and socioeconomic patterns documented in the DELICIOUS consortium. Recent analyses conducted on a larger sample of children and adolescents from the same project showed that adherence to Mediterranean diet principles, regular breakfast habits, and family meal frequency were positively associated with higher PA levels, whereas excessive screen time and higher consumption of ultra-processed foods were linked to lower activity and higher obesity risk. Importantly, these studies also identified parental education as a key socioeconomic determinant, influencing both children’s dietary quality and physical activity behaviors (64, 65). Together, these findings highlight the interplay between diet, lifestyle, and socioeconomic context, suggesting that the variability observed across countries in the present intervention may, in part, reflect such background factors, where children from lower socioeconomic status or less health-promoting environments likely showed smaller improvements despite comparable exposure. Genetic factors, although not directly measured in this study, also play a critical role in determining height and weight growth patterns and their responsiveness to lifestyle interventions. Previous research has demonstrated substantial genetic contributions to body size and growth trajectories, which could partly explain the observed country-specific anthropometric profiles (47). Additionally, socioeconomic and educational disparities across these countries might affect nutritional knowledge, dietary habits, and overall health behaviors, further influencing body composition outcomes (10, 11, 20, 21). Thus, the observed anthropometric variations among Mediterranean countries, either at baseline or in response to the PA intervention program, likely represent a complex interplay of lifestyle, genetic, environmental, and socio-cultural determinants.

Collectively, these findings underscore the difficulty of addressing childhood obesity through single-component PA interventions alone. Cultural, dietary, and behavioral factors critically mediate anthropometric responses (10, 66), reinforcing the need for comprehensive, context-specific strategies. Evidence indicates that multi-component interventions simultaneously targeting energy expenditure through PA and energy intake via dietary education are more effective for obesity prevention and management than single-domain approaches (67, 68). Such integrated programs foster long-term behavioral change by addressing physical activity patterns, diet, environment, and education together, promoting sustainable improvements in health and body composition (69, 70). Where feasible, combining structured PA with nutritional education, environmental supports, and balanced canteen meals aligned with Mediterranean diet principles appears essential, especially for populations showing limited responsiveness to PA alone. Nonetheless, implementation of multi-component programs in real-world school settings often faces logistical and resource constraints, underscoring the need for alternative yet holistic solutions. In this context, training strategies emphasizing motor variability, such as the Differential Learning (DL) approach, offer a promising complement to traditional models. By introducing purposeful movement variability, DL simultaneously stimulates neuromuscular, cardiovascular, and cognitive systems (71–73). Its motor-cognitive nature (74, 75) increases overall energy expenditure and engagement, particularly in children with overweight or obesity who face physical and psychological barriers to participating in high-intensity activities (76, 77). Embedding DL principles into standardized school programs may thus represent a cost-effective and inclusive strategy to promote healthy weight trajectories even in low-resource contexts.

### 4.2 Physical performance outcomes

Among normal-weight children, significant improvements were observed following the program in most tested fitness outcomes in Lebanon (all outcomes except sprint), Egypt (all outcomes except sprint and PU), and Portugal (all outcomes except sprint and SU), aligning with previous studies demonstrating the efficacy of school-based interventions in enhancing children’s overall fitness (24, 78). However, fewer significant improvements (only 2 to 3 out of 6 outcomes) were observed among normal-weight participants in Spain (JSW, Run) and Italy (JSW, Run, and SU). These limited improvements potentially reflect ceiling effects due to initially higher baseline fitness levels, and/or they may indicate that the intensity or training specificity of the intervention was insufficient for these particular groups (79).

Notably, children with overweight demonstrated the greatest responsiveness to the intervention across countries, particularly in Egypt where significant improvements were observed in all physical fitness outcomes. This was followed by Lebanon and Portugal (all outcomes improved except sprint), and then Italy and Spain, where four out of six outcomes showed significant improvement. Specifically, no significant changes were observedin sprint and SLJ among Italian children, nor in SLJ and SU among Spanish children. These results are consistent with previous research suggesting that children with overweight may experience greater relative fitness gains due to lower baseline fitness levels and a higher capacity for physiological adaptation to structured physical activity (29, 30, 35). For example, Piri et al. (30) reported that, following a 12-week school-based intervention, children with overweight showed superior improvements in jump height, agility, and sprint performance compared to their normal-weight peers. Similarly, Katanic et al. (37) found that a comparable 12-week physical exercise program led to greater improvements in both manipulation and total movement skills in overweight versus normal-weight preschool categories in Serbian children.

Biologically, children with overweight may benefit disproportionately from structured exercise interventions through improvements in muscular strength, cardiovascular function, insulin sensitivity, and metabolism, given their typically lower starting fitness levels and greater potential for physiological adaptation (46, 55). Furthermore, psychologically, children with overweight might exhibit heightened motivation and adherence to structured PA programs driven by their desire to improve body image, self-esteem, and peer acceptance (80, 81). This combination of biological and psychological factors can lead to enhanced engagement and performance gains, underlining the importance of targeted school-based interventions specifically designed to address the needs and characteristics of children with compromised baseline fitness levels.

In contrast, children with obesity exhibited the lowest responsiveness to the physical activity intervention. Specifically, improvements were limited to only one outcome in Egypt (endurance performance) and two outcomes in Italy, Spain, and Portugal (endurance performance and JSW). Lebanon was a notable exception, demonstrating improvements in four out of six outcomes, although still missing significant progress in sprint and SLJ. The reduced responsiveness among populations with obesity likely arises from a combination of physiological and psychological factors as well. Physiologically, excess adiposity may hinder movement efficiency, increase biomechanical strain, and elevate perceived exertion, particularly during high-intensity activities, thus limiting participation and diminishing the overall training response (32, 77). Obesity can also affect cardiovascular and muscular performance, leading to quicker fatigue and reduced tolerance to sustained or vigorous activity (76, 77, 82). Psychologically, children with obesity often often face lower perceived physical competence and heightened fear of peer judgment, which can undermine self-efficacy and participation in PA (76, 81, 83–85). Unlike children with overweight—whose motivation may be fueled by body image improvement—those with obesity frequently carry negative exercise experiences that dampen motivation and adherence (83, 85–87). These challenges highlight the need for psychologically supportive, confidence-building strategies (e.g., gradual intensity progression, individualized motivation emphasizing personal achievement over peer comparison, and enjoyment and self-efficacy environments) to sustain engagement among children with obesity (83, 87, 99–103). Furthermore, adopting a more differentiated intervention protocol, such as one guided by DL principles, may help optimize performance outcomes across weight categories. DL-based interventions improve coordination, agility, strength, and neuromuscular control by continuously varying movement parameters (intensity, speed, amplitude, and environment) thereby preventing performance plateaus and enhancing adaptation (71, 73, 88–91). Among children with normal weight and overweight, such variability helps overcome ceiling effects by introducing novel motor challenges that enhance adaptability. For children with obesity, DL’s gradual, perceptually tolerable fluctuations in intensity and movement minimize joint strain and perceived exertion while boosting cognitive engagement and enjoyment—key factors for motivation and long-term adherence (76, 92, 93). Empirical evidence indicates that DL enhances motor skills, cardiovascular, and neurophysiological markers (71, 72, 75, 89, 94, 95, 97), making it a promising addition to school-based PA models, particularly for heterogeneous groups. Nonetheless, further high-quality RCTs comparing DL-based interventions with other motor learning models [e.g., repetitive or contextual interference; (96)] are warranted.

### 4.3 Strengths and limitations

This study’s strengths lie in its large, diverse sample and cross-national comparative design, including five Mediterranean countries. Such a multicenter approach is uncommon in school-based fitness intervention research and may enhances the generalizability and cultural relevance of findings. By applying a standardized intervention protocol and using validated, culturally adaptable tools such as the IPPTP for fitness assessments (23, 43), we ensured higher consistency, objectivity, and reliability in measuring outcomes across diverse populations. Moreover, the study’s design allowed us to identify differential responses to PA across body weight categories and national contexts, offering nuanced insights often overlooked in single-country studies, as well as clear translational value for tailoring school-based interventions to the specific needs of each population.

However, several limitations warrant consideration. First, the absence of a control group limits our ability to attribute observed changes solely to the intervention, as potential internal validity threats such as maturation or history effects cannot be excluded. While we attempted to mitigate this by focusing on weight category-specific comparisons and percentage change calculations, future studies are advised to incorporate randomized controlled designs or maturity status estimations. Second, baseline heterogeneity between countries, particularly in their anthropometric values, cultural attitudes toward PA, and possible differences in intervention implementation, may have introduced site-specific biases. Although the intervention was delivered uniformly, children’s adherence, enthusiasm, and contextual influences (e.g., facilities, climate, support) likely varied and were not formally assessed. Moreover, standardized cross-site fidelity and adherence metrics (e.g., session delivery records, instructor training logs, and attendance rates) were not uniformly captured, which may have contributed to country-level variability and limits the interpretability of between-site comparisons. Although a predefined adherence threshold (absence from ≥14 sessions, ≈20%) was applied, future multicenter studies should incorporate harmonized fidelity frameworks to ensure consistent adherence monitoring across sites. Additionally, while the analyses incorporated body-weight category as a fixed factor and tested country × weight-status interactions to account for potential differences in prevalence across sites, residual variability may persist due to differences in the proportion and severity of overweight or obesity within each category (e.g., mean BMI values within the group with obesity). This limitation, common in multicenter designs involving heterogeneous populations, could have influenced between-country comparisons despite standardized analytical adjustments. Third, dietary intake and socio-economic status, key mediators of anthropometric and fitness outcomes, were not systematically measured, limiting our understanding of lifestyle factors influencing intervention effectiveness. This is particularly relevant given that the broader DELICIOUS project includes components related to diet and Mediterranean lifestyle adherence, which strongly interact with PA outcomes. Third, dietary intake and socioeconomic status, two important confounders of anthropometric and fitness outcomes, were not directly measured in the current intervention dataset. Nevertheless, all participants were drawn from public urban schools, suggesting relative socioeconomic homogeneity within countries. However, cross-country differences in socioeconomic factors and diet quality, previously described in related DELICIOUS studies (64, 65), could have contributed to the observed variability in BMI and performance outcomes. Future analyses combining PA-intervention data with these complementary dietary and SES datasets will help disentangle these contextual influences. -Moreover, no direct measures of fat distribution (e.g., waist circumference or waist-to-height ratio) nor data on habitual physical activity outside the intervention (e.g., from questionnaires or wearable devices) were collected. These parameters could have provided additional insights into body composition changes and overall activity behavior, and their absence limits interpretation of the broader lifestyle context influencing the observed outcomes. Future research would benefit from integrating these objective or questionnaire-based measures to more precisely capture both central adiposity and background activity patterns. Furthermore, the intervention duration, although aligned with an academic year, may not have been sufficient for some fitness outcomes (especially in children with obesity) to reach full adaptation. Longer-term follow-up would help determine the sustainability and progression of observed improvements. Finally, while age and sex were analytically incorporated into the BMI-for-age z-score classification, accounting for developmental and sex-specific growth differences, no separate interaction analyses by age or sex were conducted in this study. This choice was made to maintain analytical focus on body-weight status and minimize the risk of multiple-testing inflation or underpowered subgroup comparisons. It should be noted that age-related effects and their interaction with country were comprehensively examined and published separately by our team (24), providing detailed developmental insights. Nonetheless, the absence of age- or sex-specific interaction analyses in the present study remains a limitation, and future research should integrate these moderators within a unified analytical framework to refine understanding of adaptive responses across developmental stages.

Despite these limitations, the study provides critical insights into how context, weight status, and national culture interact with PA intervention responsiveness. These findings offer a valuable foundation for developing future multidomain strategies addressing both physical activity and dietary behaviors, particularly among vulnerable child populations such as those with overweight and obesity.

## 5 Practical recommendations for enhanced intervention effectiveness

Based on comparative patterns rather than causal inference, several pragmatic adaptations may support program responsiveness. For children with overweight, adding structured strength/skill components and short sprint/agility bouts could enhance broad fitness gains, particularly where baseline fitness is lower. For children with obesity, graded intensity progressions, low-impact options, and psychologically supportive strategies emphasizing personal achievement over peer comparison may foster engagement and adherence. Sites showing BMI stability or small reductions (e.g., Egypt, parts of Spain) may prioritize maintaining program fidelity, whereas settings with BMI increases (e.g., segments of Lebanon/Italy) could consider pairing PA with basic nutrition education or school-meal adjustments. A fuller, country-by-country set of suggested adaptations is provided in Supplementary Appendix S1.

## 6 Conclusion

This study highlights the importance of considering both body weight status and the national context when implementing school-based physical activity interventions in Mediterranean countries. Our findings demonstrate substantial variability in anthropometric and fitness outcomes across countries and weight categories, emphasizing the need for tailored strategies to enhance effectiveness, particularly among youth with overweight and obesity.

The observed differentiated responsiveness underscores the critical role of culturally adapted, multidomain interventions that go beyond traditional physical activity programs. In particular, children with obesity exhibit limited responsiveness to standard PA protocols, indicating the need for specialized modifications that address both physiological and psychological barriers. Future interventions should integrate structured physical activity, nutritional education, psychological support, and environmental modifications, thereby targeting both energy expenditure and energy intake.

Moreover, involving families, schools, and community stakeholders in a cohesive, supportive framework is key to fostering sustainable behavioral change. Such comprehensive and personalized approaches hold the greatest potential to achieve long-term improvements in body composition, physical performance, and overall health, ultimately supporting the well-being of children across diverse Mediterranean settings.

## Data Availability

The raw data supporting the conclusions of this article will be made available by the authors, without undue reservation.
