# Supplementary material for: The need for tailoring school-based physical activity interventions: preliminary insights into body weight and cross-country differences from the DELICIOUS project
Source: Front Public Health. 2025 Dec 18;13:1675893. doi: 10.3389/fpubh.2025.1675893 (PMC12756180; doi:10.3389/fpubh.2025.1675893)
Supplement: Supplementary file 1 [file Data_Sheet_1.docx]

**Supplementary Appendix S1: Country-specific Recommendations**

The present findings underscore the necessity of tailored adaptations and targeted enhancements of school-based PA interventions according to both national contexts and children's body weight categories. To optimize the outcomes and achieve comprehensive improvements in all tested fitness domains, we propose specific adaptations to complement the existing standardized PA intervention:

***Egypt:*** While Egyptian children generally benefited significantly across most fitness domains, the lack of improvement in sprint performance among normal-weight participants suggests the need for additional speed-oriented exercises. Future adaptations should include short-interval sprint training, reaction-time drills, and agility circuits designed specifically to develop speed and explosive movements. Integrating competitive but inclusive games emphasizing rapid movements and acceleration could also enhance motivation and enjoyment, thereby maximizing speed-related outcomes (Sutapa et al., 2021; Legarra-Gorgoñon et al., 2025).

***Spain:*** Spanish children displayed limited responsiveness, especially in strength (PU, SU) and explosive power (SLJ) among children with overweight, and strength and sprint outcomes among normal-weight children. This lack of responsiveness could, in part, be attributed to their comparatively higher baseline performance levels relative to other countries. Physical activity interventions aiming to enhance overall physical performance should incorporate structured muscular strength training component, such as bodyweight resistance exercises (e.g., PU, SU, planks), plyometric exercises (jump training), and high-intensity interval training (HIIT) sessions could prove beneficial. Incorporating progressive intensity and personalized goal, to maximise their effectiveness (Noone et al., 2024).

***Italy and Lebanon:*** In Italy and Lebanon, observed increases in BMI within normal and overweight categories underline the necessity for comprehensive interventions combining structured PA programs with nutritional education and dietary counseling. Intervention strategies should explicitly address regional dietary behaviors, promoting adherence to balanced dietary patterns, particularly Mediterranean dietary principles emphasizing fruits, vegetables, whole grains, and healthy fats (Masini et al., 2024, Ammar et al. 2025). The inclusion of practical nutritional workshops, balanced school canteen meals, and active parental involvement could further support favorable anthropometric outcomes (Al-Khudairy et al., 2017; Verjans-Janssen et al., 2018; Psaltopoulou et al., 2019). Additionally, considering the lack of improvement in sprint performance in both countries, targeted speed training activities including short-burst sprints and agility exercises could be integrated, complementing the nutritional component to holistically address body composition and performance deficits.

***Portugal:*** Portuguese normal-weight children did not significantly improve in sprint and sit-up performances. Thus, incorporating tailored exercises that specifically target abdominal strength and muscular endurance (e.g., core circuits, stability exercises) alongside short-distance sprint and reaction-time drills should be prioritized. Enhancing engagement through gamified activities and regular performance assessments could increase motivation and adherence, ultimately fostering comprehensive improvements (Sutapa et al., 2021; Legarra-Gorgoñon et al., 2025).

***Special Attention for Children with Obesity Across Countries***

Children categorized with obesity across most participating countries (Egypt, Italy, Spain, and Portugal) demonstrated limited improvements, emphasizing the need for specialized adaptations in future PA interventions. Given physiological barriers such as lower muscular strength, reduced cardiovascular endurance, and biomechanical challenges, the inclusion of lower-impact activities, progressive intensity adjustments, and tailored exercise programs designed to gradually enhance fitness capacity is recommended (Hills et al., 2011; Thivel et al., 2016; Noone et al., 2024). Aquatic exercises, low-impact aerobic activities, and modified strength-training circuits can effectively enhance participation and adherence while reducing discomfort and physical strain. Moreover, psychological barriers, including reduced self-efficacy, perceived incompetence, negative body image, and fear of peer judgment, highlight the importance of integrating psychological support and motivation-focused components. Individualized motivational strategies such as coach’s and/or peer’s verbal encouragement, which prioritize personal achievements, promote incremental goal-setting, and avoid peer comparisons, can significantly increase self-confidence and sustain engagement in physical activities (Zabinski et al., 2003; Hesketh et al., 2017; Soylu et al. 2024, Yilmaz et al. 2025, Romdhani et al. 2024, Sahli et al. 2024).

***Importance of Multi-component Approaches and/or integration of DL principles***

Beyond physical activity alone, addressing childhood overweight and obesity effectively requires comprehensive multidomain interventions simultaneously targeting both sides of the energy balance: energy expenditure (structured PA) and energy intake (balanced dietary habits). Multicomponent interventions combining structured physical exercises, nutritional education, and psychological support have consistently demonstrated higher effectiveness compared to single-component interventions (Al-Khudairy et al., 2017; Verjans-Janssen et al., 2018; Brown et al., 2019; Psaltopoulou et al., 2019). Therefore, future intervention programs should ideally integrate nutritional education sessions, structured physical activities tailored by weight category and fitness level, psychological support mechanisms, environmental modifications promoting active lifestyles, and parental or family involvement to facilitate long-term behavioral changes and sustained improvements in body composition, fitness, and overall health outcomes. However, in many real-world school environments, particularly in resource-limited or low-infrastructure settings, the full implementation of multi-component interventions may not always be feasible. In these contexts, adapting the structure of PA interventions to reflect multi-component benefits through alternative pedagogical strategies becomes crucial. One such promising approach is the integration of DL principles. Rooted in motor learning theory and neurophysiology, DL introduces purposeful movement variability and noise into the training context, thereby stimulating simultaneous neuromuscular, cardiovascular, and cognitive activation (Schöllhorn et al., 2012; Ammar et al., 2024a; Henz & Schöllhorn, 2017). By embedding cognitively demanding, non-repetitive motor tasks into PA sessions, DL-based PA interventions can enhance total energy output, promote sustained attention, and improve adherence and motivation, particularly in children with overweight or obesity who may face emotional or physiological barriers to traditional exercise. The non-linear and exploratory nature of DL also supports inclusive participation and reduces fear of failure or comparison, often reported among youth with obesity. This integrative stimulation may mimic the multidimensional benefits of combined physical, emotional, social and cognitive interventions, by promoting brain plasticity, metabolic activation, motor skill development, and psycho-emotional engagement. Nevertheless, future comparative studies are warranted to determine the most effective PA intervention models for school-based settings, considering not only their impact but also their feasibility, scalability, and resource demands across diverse educational and socio-economic contexts.
